# The changing landscape of multimodality imaging in congenital heart disease: white paper

**DOI:** 10.1093/ehjimp/qyaf116

**Published:** 2025-09-23

**Authors:** G Di Salvo, J Sabatino, S V Babu-Narayan, A Arvanitaki, B Bonello, A Van De Bruaene, M Cantinotti, L Dos Subira, F Raimondi, T Bharucha, R Enache, G Greil, H Latus, M Bhat, E Valsangiacomo, B Cosyns, S E Petersen, V Delgado, H Grotenhuis, M Brida, Jolien W Roos-Hesselink

**Affiliations:** Department of Women’s and Children’s Health, Pediatric Cardiology and Adult Congenital Heart Disease, University of Padua, Via Giustiniani, Padova 3 - 35128, Italy; Department of Women’s and Children’s Health, Pediatric Cardiology and Adult Congenital Heart Disease, University of Padua, Via Giustiniani, Padova 3 - 35128, Italy; Department of Adult Congenital Heart Disease, Royal Brompton Hospital, London, United Kingdom; Adult Congenital Heart Centre and National Centre for Pulmonary Hypertension, Royal Brompton and Harefield Hospitals, Guy's and St Thomas’s NHS Foundation Trust, Imperial College, London, United Kingdom; Pediatric Cardiology, Great Ormond Street Hospital, Great Ormond street, London WC1N 3JH, UK; Great Ormond Street Institute of Child Health, University College of London, 30 Guilford street, London WC1N 1EH, UK; Congenital Cardiology, University Hospitals Leuven, Leuven, Belgium; Department of Pediatric Cardiology and GUCH Unit, Fondazione CNR Regione Toscana G Monasterio, Pisa, Italy; Adult Congenital Heart Disease Unit, Vall d'Hebron University Hospital, Barcelona, Spain; Pediatric and Adult Congenital Heart Disease, pope Giovanni XXIII Hospital, Bergamo, Italy; Department of Paediatric Cardiology, University Hospital Southampton NHS Foundation Trust, Southampton, UK; Pediatric Cardiology Department, Prof. Dr. C.C. Iliescu Emergency Institute for Cardiovascular Diseases, Bucharest, Romania; Cardiology Department, Carol Davila University of Medicine and Pharmacy, Bucharest, Romania; Department of Pediatrics, University of Texas Southwestern Medical Center, Dallas, TX, USA; Pediatric Heart Center, University Children's Hospital, Feulgenstrasse 12, Giessen D-35392, Germany; Department of Pediatric Cardiology, Pediatric Heart Center, Avd 67 Skåne University Hospital in Lund, Lund SE-221-85, Sweden; Division of Pediatric Cardiology, University Children's Hospital, Steinwiesstrasse 75, Zurich 8032, Switzerland; Department of Cardiology, CHVZ (Centrum Voor Hart en Vaatziekten), Universitair Ziekenhuis, 101 Laarbeeklaan, B Brussels, Belgium; William Harvey Research Institute, NIHR Barts Biomedical Research Centre, Queen Mary University London, Charterhouse Square, London EC1M 6BQ, UK; Barts Heart Centre, St Bartholomew’s Hospital, Barts Health NHS Trust, West Smithfield, London EC1A 7BE, UK; Cardiology, Hospital University Germans Trias i Pujol, Badalona, Spain; Heart Institute, Institute for Health Science Research Germans Trias i Pujol (IGTP), Badalona, Spain; Department of Pediatric Cardiology, Wilhelmina Children's Hospital, University Medical Center Utrecht, Utrecht, the Netherlands; Adult Congenital Heart Centre and National Centre for Pulmonary Hypertension, Royal Brompton & Harefield Hospitals, Guys & St Thomas’s NHS Trust and National Heart and Lung Institute, Imperial College, Sydney Street, London SW3 6NP, UK; Department of Cardiology, Thorax Centre, Erasmus Medical Centre, Rotterdam, The Netherlands

**Keywords:** congenital heart disease, multimodality imaging, life-long care

## Abstract

The congenital heart disease (CHD) population has seen substantial growth due to advancements in paediatric cardiac care, cardiothoracic surgery, anaesthesia, and intensive care. Although improved surgical interventions have enhanced survival rates for even the most severe defects, such as hypoplastic left heart syndrome, most CHD patients remain palliated rather than cured, requiring lifelong expert care. With increasing survival, new challenges emerge in managing the ageing CHD population, particularly regarding changing medical, psychosocial needs, and the evolving epidemiology of CHD. Imaging plays a pivotal role in CHD care, offering precise anatomical and physiological insights necessary for effective management and risk stratification. The European Association of Cardiovascular Imaging (EACVI) established a shared interest group (SIG) to improve imaging standardization, promote education, and foster research for CHD patients. Multimodality imaging, including echocardiography, cardiovascular magnetic resonance (CMR), and computed tomography (CT), is critical in guiding interventions and improving outcomes. The SIG also addresses gaps in education, research, and global healthcare disparities, ensuring a collaborative and systematic approach to CHD care. Advances in imaging technology, genetic research, and tissue engineering hold promise for further improving the longevity and quality of life for CHD patients in the future.

## Graphical Abstract

**Graphical Abstract:**
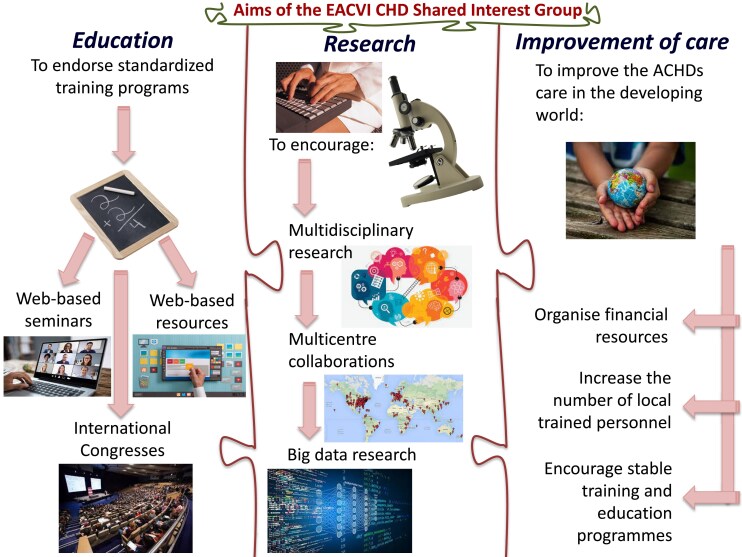

## Introduction

The congenital heart disease (CHD) population has considerably grown in recent decades, due to tremendous advances in paediatric cardiac care, cardiothoracic surgery, anaesthesia and intensive care.^1,2^ Even for severe defects, such as hypoplastic left heart syndrome, currently available surgical options enable good chances of survival to adulthood.

Notwithstanding, the improved life expectancy, the majority of CHD patients cannot be completely cured and instead are often palliated. Surgery allows repair rather than correction. CHD is a lifelong condition necessitating life-long expert care to ensure optimal longevity and quality of life is achieved including through timely interventions. Improved survival in CHD presents new challenges in the care of these patients, especially in adulthood, related to the growing and ageing adult CHD population, with changing priorities and medical and psychosocial needs.^3^

Recently, the European Association of Cardiovascular Imaging (EACVI), recognizing the unique nature of caring for CHD patients, established firstly a task force that more recently evolved in a Shared Interest Group (SIG), consisting of experts from all parts of Europe and with expertise in the different imaging modalities and in CHD cardiology. The scope of the new SIG is to appraise the current status of care for CHD patients across the globe with the focus on imaging. Goals are to improve the standardization of cardiovascular imaging (CVI) practice for CHD patients, highlight major issues, challenges and imaging priorities, and to promote research to improve clinical practice including by encouraging multicentre studies. The CHD SIG should support knowledge-sharing and interaction with other EACVI task forces and SIGs, promote education and training with the overall aim to reduce mortality and morbidity and improve quality of life in patients with CHD.

### The changing pattern of CHD epidemiology

Out of an estimated 150 million live births per year across the world, about 1.35 million are affected by CHD.^1^ In developing countries (∼85% of the world's population), the access to appropriate CHD care is limited; as a result, thousands of children die each year of CHD.

In western countries more than 90% of children with CHD now survive into adulthood.^2^

The epidemiological consequence of the success of paediatric CHD care is that the adult CHD population has grown steadily in the past few decades, even considering those categories formerly characterized by high mortality in infancy, such as children born with hypoplastic left heart syndrome.

As a result, the adult CHD population now outnumbers the paediatric CHD population in most Western countries, with a population that continues to increase. In Europe, among 800 million people, ∼2.3 million are adult CHD patients and 1.9 million are paediatric patients with CHD; therefore, adult with CHD represent about 60% of the total CHD population.^3^ In addition to already outweighing the population of children with CHD the number of adults is growing and care of the elderly CHD patients or adults with multiple long-term adult general medical conditions is an increasingly frequent requirement.

Although the prevalence of CHD at birth is comparable between males and females, in adults the proportion of women is higher. Correspondingly, CHD mortality was demonstrated to be higher in males than in females in a large European survey.^4^ Among adult people with CHD, males are more likely to die from late complications such as infective endocarditis, arrhythmia, and aortic diseases.^5^ On the contrary, pulmonary hypertension affects females more frequently.^6^

Even though many CHD patients may lead a functionally normal life, reinterventions are often required, and mortality is undoubtedly higher in CHD patients in comparison with the general population.^7^

Heart failure has emerged as the leading cause of death in this population, resulting from residual structural problems (such as valvar insufficiency), cardiac repair sequelae (e.g. residual shunts or valve dysfunction), or the underlying congenital disease itself causing progressive pressure and or volume load on the ventricle (such as abnormal loading of the systemic right ventricle).^8^ Arrhythmias (both atrial and ventricular) are also prevalent in adult CHD patients, resulting in higher morbidity and the added risk of (sudden) cardiac death.^9^

Furthermore, adult CHD patients often present with other problems, including an increased burden of thromboembolism and stroke, exercise intolerance, reduced peak oxygen consumption and a high risk of endocarditis. Also, progressive hepatic fibrosis occurs in specific groups, especially those with higher systemic venous pressures such as after Fontan completion.^9,10^ Finally, patients with CHD want to live an unrestricted life, but sports participation and pregnancy may carry high risks, especially for some specific groups. Women with CHD who become pregnant encounter more frequent adverse cardiovascular events, and have an elevated risk of obstetric and foetal complications.^11^

These issues emphasize the need for detailed multi-modality imaging assessment for serial surveillance in patient care. This is not only for the precise anatomy of the specific congenital heart lesion being imaged but also to understand the current pathophysiology in order to influence the timing of proactive intervention and to optimize risk stratification.

Thus, awareness of the changing profile of the contemporary CHD population, that includes knowledge of previous eras of now abandoned surgical approaches, emerging new approaches being applied in the current paediatric population and that accounts for demographic progression, disease severity, and solicited healthcare sources, is fundamental in understanding needs and allocating novel models of care targeted to the CHD population.

### Imaging the patient with CHD: the central role of multimodality imaging

From the earliest images of cardiac catheterization by Forssman in 1929, advances in cardiovascular imaging have been an excellent vehicle for the success of surgical procedures for CHDs. What was originally a mainly X-ray-based imaging approach has evolved into advanced multi-modality imaging techniques, which include two- (2D) and three-dimensional (3D) echocardiography, cardiovascular magnetic resonance imaging (CMR), cardiac tomography (CT) angiography, and using cardiovascular image reconstructions for 3D printing and virtual reality for guiding invasive surgical, structural interventional and electrophysiology procedures.

Nowadays, multi-modality imaging plays a fundamental role in the precise depiction of the defect’s anatomy, such as diagnosing myocardial anomalies and intra-cardiac shunts and the associated (patho)physiology, including ventricular and valve (dys)function, and routinely is used to guide management such as indications or timing of intervention.^12^

The appropriate imaging modality choice should take into consideration both the risks for the patient and the test’s ability to provide a given answer for a specific diagnosis.^13,14^ Availability of the specific technology, skills of the available personnel and financial constraints are possible limiting factors.

Echocardiography is the primary imaging modality to be used in most situations, included in routine surveillance with intervals varying according to the current pathophysiology, and is often able to provide a lot of essential information required for the specific diagnosis and management of CHD.^13^ Stress echocardiography is used selectively by clinical indication. Transoesophageal echocardiography should be routinely available for CHD periprocedural guidance, in both cardiac theatres and the catheter catheterization laboratory.

More recent advanced echocardiography modalities, such as speckle tracking echocardiography and 3D echocardiography may be helpful to detect subtle myocardial dysfunction, before overt heart failure occurs, and may be of use for a finer surveillance and risk stratification of CHD patients.^13^ Specific alterations in haemodynamics and fluid-dynamics are now within the scope of recently developed techniques of advanced echocardiography, able to assess the shape and position of intracardiac vortices; these may be of value for better definition of CHD physiology in the near future.^15^ High frame rate imaging represents an exciting avenue to assess myocardial stiffness and diastolic properties in selected CHD subgroups.^16^

CMR is a well-established and important diagnostic technique and part of guideline-directed follow-up of CHD patients. It can provide precise parameters needed for clinical decision-making; among those, quantified right (and left) ventricular (RV) volumes, ejection fraction EF, degree of valvar regurgitation, and Qp:Qs in shunt lesions, tissue characterization in patients with ventricular dysfunction or clinical symptoms after CHD surgery involving reimplantation of the coronary arteries, or at selective attendances of patients with specific ACHD lesions where adult ventricular scar status has been shown to predict outcomes,^13^ for relationships of the heart and great vessels to the mid-sternum prior to redo sternotomy and finally for evaluation of extracardiac vascular and other lesions, or the heart itself where echocardiographic windows are poor. CMR 4D flow and 3D cine data sets allow offline reconstruction and quantification in any imaging plane. If both CMR and CT are appropriate, CMR is usually preferred over CT to limit the cumulative dose of radiation for CHD patients and to avoid iodinated contrast risking kidney toxicity.^13^ CT can answer many of the questions CMR is used to answer and becomes the modality of choice in case of significant metallic artefacts or when higher spatial resolution is desirable, such as for intramural course of coronary anomalies, small collateral arteries or veins, arteriovenous malformations or for coronary artery disease diagnosis.

Finally, nuclear imaging is generally used for the evaluation of myocardial stress perfusion, when appropriate stress echocardiography (exercise/dobutamine) or stress CMR (vasodilator/dobutamine/exercise) are not an option. However, this technique also has the disadvantage of radiation exposure and is relatively expensive. PET-CT might be of additional value in the case of (suspicion of) endocarditis, especially for prosthetic heart valves.

Cardiac segmentation of CT or MRI data for 3D printing or virtual/augmented reality imaging can be used to produce a replica of the individual patient’s heart anatomy to enable detailed pre-surgical or interventional planning in more complex CHD cases. Personalized 3D heart prints may be helpful tools for patient counselling, consenting, simulation and trainee education. Virtual and augmented reality imaging enable the user to interact with the 3D cardiovascular reconstructions in a true 3-dimensional way without the need for fabrication (3D printing).

### Aims of the EACVI CHD SIG

The overall aim of the EACVI CHD SIG is to provide excellence in the care of paediatrics and adults with CHD through optimal imaging assessment by appropriately skilled staff across Europe and the world. This will be achieved by promoting education, research, and collaboration with other societies on a global and inclusive scale.

#### Education

The EACVI CHD SIG aims to improve the quality of care by endorsing, supporting and organizing educational programmes in the field of (multi-modality) imaging in CHD.

Although it is widely recognized that caring for CHD patients requires specific training, the actual fellowship programs differ between European countries, and are currently absent or poorly defined in many countries. Moreover, the priority in funding programmes is low for CHD, when compared with other cardiology subspecialties. CHD care is often viewed as being expensive and with current health and care organizational structures as non-income generating with respect to other ‘traditional’ cardiovascular diseases.

Another issue regarding CHD education is that despite the need for CHD cardiologists based on burden of disease, cardiology fellows may not be willing to prolong their training with an CHD subspecialty fellowship which is typically required, or perceive it as a high-risk subspecialty cardiology choice, given CHD consultant position availability may not be easy to forecast.

Therefore, the EACVI SIG is seeking to support the CHD cultural diffusion and awareness and to endorse and develop standardized training programmes in Europe with a focus on imaging, with the goal of establishing official recognition. Hospital management and cardiology directors should be aware that the growth of an CHD programmes (in terms of patient numbers and complexity of care) requires an equal expansion in care facilities, with dedicated and specialized personnel, and adequate resources.

Specific ESC/EACVI accreditations in CHD echocardiography and CHD CMR, respectively, are already available. Our objective is to improve further and to disseminate the standardization of CHD imaging training and practice.

Accordingly, web-based seminars and resources will be organized to help deliver CHD education more widely and available to all countries in Europe. In addition, multimodality imaging (MMI) training programmes will be introduced and advanced imaging modality training opportunities in high-volume centres.

Specific sessions dedicated to CHD imaging or MMI during International Congresses, such as EACVI, EuroACHD and ESC, require further encouragement and dissemination.

Finally, ‘lesion-specific’ MMI education papers will be published by those with practical experience in order to help educate and support others with implementation. Such recommended lesion-specific protocols would help standardize core imaging data sets and enable future multicentre research.

#### Research

The EACVI CHD SIG aims to establish guidelines for imaging patients with CHD.

MRI-guided CHD intervention and MR-augmented catheterization are emerging approaches for more accurate evaluation in complex CHD.^17^ The use of 3D image reconstructions or virtual reality allows for the development of new devices and helps define suitability for CHD invasive procedures.^18^ Additionally, intraprocedural integration of 3D reconstructed cardiac imaging data can facilitate remote navigation for electrophysiology treatments.^18^ PET CT or PET MRI can also be used to assess the potential role of microcalcification diagnosis in predicting progressive aortopathy.^19^

Despite the growth in number and impact of research publications on CHDs over the last years, numerous questions are still to be answered. Firstly, the current CHD research modality mainly comprises single-centre or at the most collaborative research, with only recent and very few papers, embracing multidisciplinary, and MMI data and/or big data. A recent development is the recognition of a European Reference Network for CHD, within Guard Heart.

Clinical studies with important endpoints such as mortality or major adverse cardiovascular events, large enough to have enough endpoints and with sufficient granularity to be meaningful, are challenging in these rare CHD conditions, given their heterogeneity (even within lesion).

The heterogeneity of CHD makes it challenging to perform large studies for some diagnostic groups and to produce results in time to be generalizable to the cohort of CHD patients that will be contemporary by the time the study ends. Studies on rare defects such as these are clearly crucial. In this context, multicentre studies are warranted and appropriate imaging secondary endpoints can have a vital role. Research studies that collaborate across multiple centres will benefit from wider available training and the implementation of standardized lesion-specific core imaging data sets, such as those already recommended in international guidelines for CHD echocardiography.^20^

Divergent nomenclature and classifications across institutions and nations could confound multicentre research attempts and therefore it is important to establish clear international definitions.

Last but not least, funding allocation and research facilities are often limited in this field, creating further barriers to CHD research.

The EACVI CHD SIG intends to promote multicenter research and to solicit a multidisciplinary approach that involves collaboration among paediatric cardiologists, surgeons, adult cardiologists, interventional cardiologists, anaesthesiologists, paediatricians, radiologists, critical care medicine physicians and other specialists.

In addition to the attention for rare defects, we encourage the progress of big data research within and beyond the CHD research community, via open platforms that may ease collaborations among disciplines. Finally, engaging and involving patients is also an important priority, with patient-reported outcomes having lately become crucial end points in clinical trials.^21^

#### Improvement of care

The aim of the EACVI CHD SIG is to establish close links with other groups/societies dealing with imaging adult cardiac disease. CHD care has nowadays reached unprecedented successes with results not even conceivable only a few decades ago. However, in this ageing group population, adult cardiac diseases occur on the top of the congenital anomaly and a close collaboration within imaging experts for adult cardiology is essential. Moreover, parts of the world still require basic access to CHD care to be established.

It is now time to encourage a crucial paradigm shift in the CHD care to facilitate the global and inclusive treatment improvement. This can only come through a systematic re-organization of healthcare providers worldwide.

Thus, some of the aspirations of this EACVI CHD SIG are to influence change towards improving health and care system financial resources, including increasing the number of local trained personnel, to encourage the establishment of stable, funded training and education programmes to support good ACHD health care throughout Europe as well as in developing countries.

## Conclusions and future perspectives

Cardiovascular MMI is now the key to improving the diagnosis and management of patients with CHD. Collaboration between the different cardiac subspecialties and across countries is warranted.

Advances in the next decade will probably see the dissemination of newer technologies necessary to treat the full spectrum of CHD, improved precision and effectiveness of surgery and cardiac catheterization procedures, and reduced long-term extracardiac morbidities consequent to living with CHD. Genetic and epigenetic studies will further individualize CHD care, identifying family members at risk and providing counselling in cases of heritable disease. Tissue engineering and stem cells research may soon provide new valves and vascular structures originating from the patients’ own cells, that do not degenerate as quickly, consequently reducing the number of interventions a patient is recommended lifelong, and providing targeted cell therapies to improve myocardial function in ischaemic and non-ischaemic cardiomyopathies.

Parallel advances in imaging technologies, such as real-time imaging, higher spatial resolution, multimodality image fusion to guide procedures, and artificial intelligence^22^ enabled faster and more automated imaging acquisition and reporting, can be expected. These will allow more accurate approaches to cardiovascular surgery, catheter-based intervention, treatment of arrhythmia, and prevention of sudden cardiac death.

These are only a few of likely many future developments. Without any doubt, there is still much work to be done. The pioneering and successful history of CHD, which can claim many major landmarks in the progress of cardiology, gives us reasons to hope for a promising future.

## Data Availability

Not applicable.

## Lead author biography

Prof. Giovanni Di Salvo is Director of Pediatric Cardiology and Adult Congenital Cardiology Program at the University Hospital of Padova, Italy. He is an internationally recognized expert in CHD. Prof. Di Salvo plays a leading role in CHD education across Europe, coordinating high-impact training initiatives and courses. His research focuses on advanced echocardiographic methods.
